# *Vibrio coralliilyticus* Search Patterns across an Oxygen Gradient

**DOI:** 10.1371/journal.pone.0067975

**Published:** 2013-07-10

**Authors:** Karina M. Winn, David G. Bourne, James G. Mitchell

**Affiliations:** 1 School of Biological Science, Flinders University, Adelaide, South Australia, Australia; 2 Centre for Marine Microbiology and Genetics, Australian Institute of Marine Science, Townsville, Queensland, Australia; Universidade Federal do Rio de Janeiro, Brazil

## Abstract

The coral pathogen, *Vibrio coralliilyticus* shows specific chemotactic search pattern preference for oxic and anoxic conditions, with the newly identified 3-step flick search pattern dominating the patterns used in oxic conditions. We analyzed motile *V. coralliilyticus* cells for behavioral changes with varying oxygen concentrations to mimic the natural coral environment exhibited during light and dark conditions. Results showed that 3-step flicks were 1.4× (P = 0.006) more likely to occur in oxic conditions than anoxic conditions with mean values of 18 flicks (95% CI = 0.4, n = 53) identified in oxic regions compared to 13 (95% CI = 0.5, n = 38) at anoxic areas. In contrast, run and reverse search patterns were more frequent in anoxic regions with a mean value of 15 (95% CI = 0.7, n = 46), compared to a mean value of 10 (95% CI = 0.8, n = 29) at oxic regions. Straight swimming search patterns remained similar across oxic and anoxic regions with a mean value of 13 (95% CI = 0.7, n = oxic: 13, anoxic: 14). *V. coralliilyticus* remained motile in oxic and anoxic conditions, however, the 3-step flick search pattern occurred in oxic conditions. This result provides an approach to further investigate the 3-step flick.

## Introduction

Bacteria use chemotaxis and motility search patterns to position themselves in chemical gradients, locate nutrient sources and initiate pathogenesis [1], [2]. These search patterns include run and reverse, run and tumble, straight swimming and the recently described, the 3-step run, reverse and flick [2], [3], [4], [5]. The majority of evidence suggests that only one search strategy is adopted by a single bacterial species [6], [7], with marine bacteria suggested to adopt a ‘back and forth’ swimming behavior pattern, known as a run and reverse, utilizing turbulence-induced shear in the ocean [8]. While the interactions of the run and reverse pattern with its environment is clear, how the 3-step flick pattern, henceforth ‘the flick’, which is a cyclic motion: forward, reverse, flick and repeat [5], [9] interacts with the physical environment is less clear. What is known so far is that by introducing a consistent directional change, the flick allows species such as *V. alginolyticus* to access nutrient patches quicker than bacteria using the run and tumble strategy such as *Escherichia coli* [5]. The key trait of the flick search strategy seems to be a combination of the run and reverse strategy, where the 180° backtracking gives bacteria traveling down a gradient an opportunity to re-exploit nutrient patches found moments earlier, with the run and tumble strategy uses frequent reorientation to find new nutrient sources [3].

Pathogenic and symbiotic *Vibrio* species, including the coral pathogen *V. coralliilyticus*, are reliant on chemotaxis to invade and colonize host species. Abundant in the coral mucus [10], [11] and the microbial community of diseased corals [12], [13], *Vibrios* alter the microbial metabolism in corals [14]. *V. coralliilyticus* causes the bleaching and tissue lysis of corals at temperatures greater than 25°C, and is further implicated in the disease of other marine organisms including bivalves, during winter months when temperatures are lower [15]. Meron *et al.* [16] demonstrated that the flagellum in *V. coralliilyticus* is critical for infection, including the adhesion to the corals and chemotaxis towards coral mucus.

During coral infection, *V. coralliilyticus* must move through the surrounding seawater, the coral mucus surface layer and into the coral tissue cells for establishment and growth. This infection route, indicative of most coral pathogens, may illustrate unique search pattern strategies to assist in moving through numerous environments of differing viscosity and nutrient complexity. Local marine environments and conditions are constantly changing due to turbulence [9]. Changes in conditions and nutrient concentrations occur diurnally and nocturnally [17]. Oxygen saturation levels are reduced in coral tissue after being exposed to the darkness, with records showing that coral tissues have been shown to have <2% oxygen saturation after 5 minutes in the dark [17]. In contrast, during light conditions coral tissues exhibit up to 250% oxygen saturation [17]. Furthermore, work by Kühl *et al.* [17] showed that oxygen levels remained steady in the surrounding water in both light and low-light conditions, however these levels have shown to differ during light and low-light conditions in the 0.1–1 mm layer on the coral tissue surface as well as inside the coral tissue [17]. This study examines the chemotactic search pattern changes of *V. coralliilyticus* in response to fluctuating oxygen levels. This will provide insight into how *V. coralliilyticus* behaves in fluctuating oxygen levels from the surrounding seawater, surface mucus layer or boundary layer and the coral tissue.

## Materials and Methods

### Bacterial Culture and Growth

The single, polar flagellated *V. coralliilyticus* type strain, LMG20984 was used for all experiments. Pure cultures were stored at −80°C in 15% glycerol stock solutions. Strains grew on Thiosulphate citrate bile salts sucrose agar media (Sigma Aldrich) overnight at 28°C. Liquid cultures were prepared in 30 mL of Luria-Bertani broth (Sigma Aldrich), incubated 16 hrs overnight at 28°C with 160 rpm shaking and harvested during exponential growth, diluted with LB to 1/10 to adjust cell density to OD 0.1±0.01, and visualized within 1 minute.

### Microscope Observation Chamber and Microscope Video Analysis

**Figure 1:**
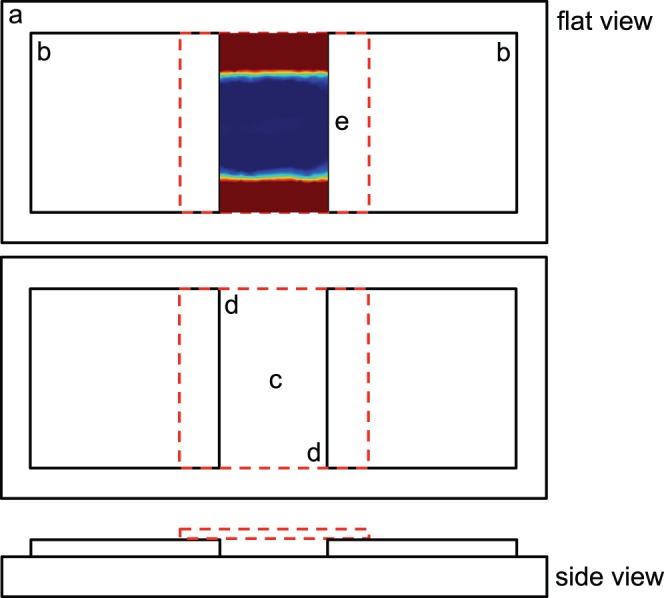
The measurement locations within the observation chamber. (a) Microscope slide, (b) cover slips, (c) center recordings, (d) edge recordings. A 3D numerical model depicts how oxygen diffuses from the top and the bottom of the coverslip labelled (e). Red depicts high oxygen concentrations with a vertical length of 5250 µm, whilst the blue depicts low oxygen concentrations. The chamber has a vertical length of 26,250 µm. The red dashed line illustrates the chamber cover, which sits on top of two cover slips.

A volume of 50 µL of cell suspension was added to an observation chamber constructed from two glass coverslips on a glass slide and covered with a third coverslip (Fig. 1). The distance between the glass slide and coverslip was 0.17 mm, with a channel width of 15 mm. Swimming cells were observed at the edge and center of the coverslip, midway between the coverslip and the microscope slide. The oxygen distribution was approximated by modeling, which illustrated that oxygen levels began to deplete within millimeters from the edge of the observation chamber. Additionally, cells were also observed at the coverslip surface. Within the microscope observation chamber, measurements were made mid chamber within 2 mm of the coverslip edge and center (Fig. 1). Swimming *V. coralliilyticus* cells were observed under dark-field, 200× magnification video-microscopy (AxioCamMrc5 camera, Carl Zeiss AxioSkop microscope) with the field of view at mid-depth in the microscope-slide chamber. The video image was recorded continuously for 10 seconds at 2.4 frames s^−1^ using a time-lapse module (Carl Zeiss AxioVision 4). Search strategies of *V. coralliilyticus* were recorded and plotted across spatial gradients. Cell swimming paths of *V. coralliilyticus* were measured frame-by-frame and traced off the computer screen using overhead transparencies. Cell paths were defined as in Barbara and Mitchell [18], with speed and turn angles calculated according to Barbara and Mitchell [19]. To ensure that all patterns could potentially include a complete flick, only cells visible for at least 5 frames were used (Fig. S1).

### Oxic to Anoxic Condition Transect Line Experiments

A volume of 50 µL of 1/10 dilution of overnight culture was added to the created observation chamber. Triplicate transects were collected ranging from the oxic to the anoxic center of the observation chamber. Each transect consisted of 40 square eyepiece quadrats of 172 by 172 µm placed next to each other. The transect line spanned from the oxic to anoxic regions across 6,880 µm. At each quadrat a 10 second, 49-frame video was recorded. All distinguishable cells in each eyepiece quadrant video were tracked and analyzed. Cells were considered distinguishable if they remained in focus for at least 5 frames. All distinguishable cell trajectories were classified into one of three search patterns: run and reverse, straight swimming or 3-step flick (Table S1). Each trajectory was then measured and the swimming speed was calculated.

### Aerobic and Anaerobic Culture Control Samples

Cell cultures and the headspaces were bubbled with either pure air or nitrogen for 10 minutes, and grown in conditions as mentioned above. Methylene blue and resazurin indicators (Sigma-Aldrich) were used to confirm whether a solution was saturated throughout the experiment [6], [20], [21], [22]. Methylene blue produced a blue/green color in oxygen-saturated cultures, where oxygen levels were above 0.5%, whereas resazurin produced a bright pink color in nitrogen-saturated cultures, where oxygen levels were below 0.1%. At values between 0.1 and 0.5%, a blue/violet color was produced [6]. For gas experiments, the slide preparation including the construction of observation chambers, the opening of culture bottles and the transfer of liquid culture onto the slides were all prepared within gas filled plastic bags to minimize any changes to saturated cultures. In addition, cell-free media broth used for dilutions was also bubbled with air or nitrogen. Slides were viewed immediately and video recordings were taken within 1–2 minutes after the removal from the gas-filled bag. Previous testing showed that cultures remained colored, either green-blue or pink for aerobic or anaerobic tests respectively, for an average time of 180 seconds. This was calculated by measuring how long the color held when viewing the cultures under the microscope. Ten replicates were measured with color holding values ranging from 160 to 220 seconds. To remain certain that *V. coralliilyticus* was being viewed in oxic or anoxic conditions all videos were recorded well before the lower value in this time window.

### *Vibrio coralliilyticus* Search Pattern Changes Over Time

Microscope videos in oxic and anoxic conditions were collected at 5 minute intervals between 0 and 25 mins to measure the behavioral changes of *V. coralliilyticus* over time. Slides were prepared as stated above. New observation chambers were prepared for each oxygen condition of the timed experiment, which was run in triplicate (Table S2).

### Mathematical Modeling

**Figure 2:**
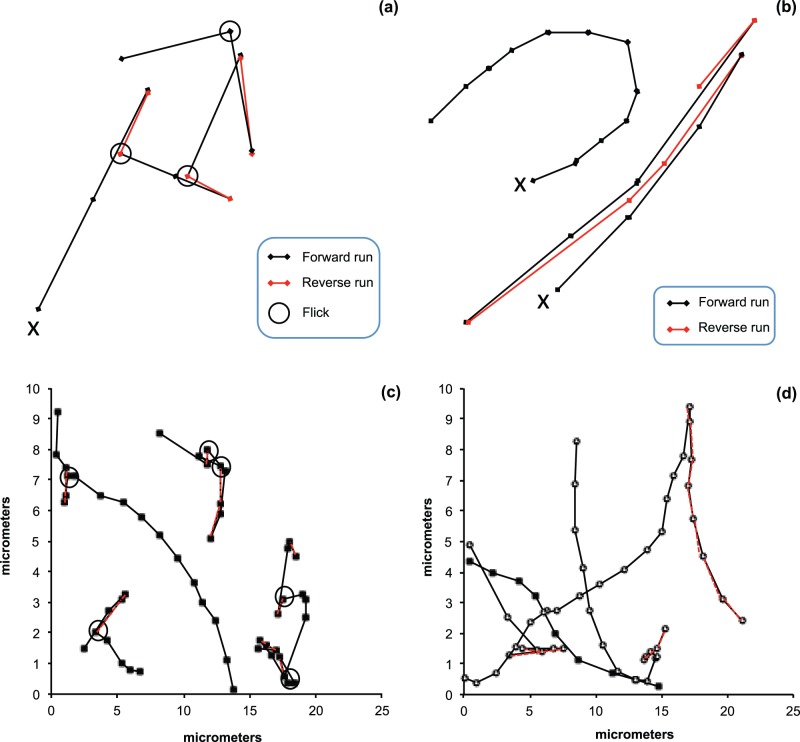
Schematic and observed chemotactic search patterns of *V. coralliilyticus*. The search pattern path starts at X; (a) cyclic 3-step flick search pattern, shown as a forward run, a reverse; seen as a 180° reorientation, and a 90° flick of the flagellum and repeat; (b) run and reverse and swimming search pattern. Run and reverse search patterns are characteristic of a 180° reorientation and reversal. Straight swimming search patterns shows no reversals; (c) 3-step flick search pattern trajectories (closed squares) collected from *V. coralliilyticus* overnight cultures collected from the oxic region of the observation chamber. For clarity, the open circles mark the 90° flicking events and the red-dashed lines indicate the 180° reversals; (d) search pattern trajectories collected from *V. coralliilyticus* overnight cultures collected from the anoxic region of the observation chamber, the search patterns exhibited here are run and reverses (open circles) and straight swimming (closed squares).



Numerical simulations of oxygen diffusion in the presence of bacteria were produced using the MatLab-derived COMSOL Multiphysics with the shown geometries using transport of dilute species with convection and diffusion models (Fig. 2). Previous studies have illustrated multiple ways in which this model has been used [23], [24], [25]. Dilute species transport was represented by.
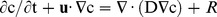
(1)where c is concentration (mol/m^3^), D is the diffusion coefficient (m^2^/s), *R* is the reaction rate for the species (mol/(m^3^s)) and in this case is used to signify flux, **u** is the vector velocity (m/s). Low Reynolds number, incompressible flows are characterized by

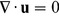
(2)However, a modeled chamber depth of 0.2 mm and isothermal conditions damped convective flow and left only molecular diffusion. Equations (1) and (2) were coupled in the MatLab-derived COMSOL Multiphysics 4.0a as a finite element 3 dimensional, free tetrahedron mesh model. The minimum mesh size was 10^−7^ m.

### Statistical Analysis

All cell trajectories were classified into search patterns and the results were analyzed using SPSS Statistics (version 19.0). Independent sample t-tests were utilized to calculate P values to establish whether flick or non-flick search patterns differ in total numbers or speed across the two observation chamber locations. T-tests were also used to determine any statistical differences in search pattern behavior during the time series.

## Results

### *Vibrio coralliilyticus* Search Pattern Preferences in Oxic and Anoxic Conditions

*V. coralliilyticus* search paths were identified and classified into three search strategies, straight swimming, run and reverse and the 3-step flick (Fig. 2). In oxic areas of the observation chamber, 53 flicks, 29 run and reverses and 13 straight swimming search patterns with mean speeds of 17 µm s^−1^ (95% Confidence Interval (CI) = 2.0, n = 53), 21 µm s^−1^ (95% CI = 4.1, n = 29) and 21 µm s^−1^ (95% CI = 8.4, n = 13) respectively, were observed (Table 1). In anoxic areas of the observation chamber, 38 flicks, 46 run and reverses and 14 straight swimming search patterns with mean speeds of 20 µm s^−1^ (95% CI = 2.6, n = 38), 20 µm s^−1^ (95% CI = 2.5, n = 46) and 18 µm s^−1^ (95% CI = 5.5, n = 14) respectively, were observed (Table 1). Flick search patterns in oxic areas of the observation viewing chamber, namely the edge, were 1.4 times more frequent in number than at anoxic, or center areas of the viewing chamber (P  = 0.006) (95% CI = 1.5, n = oxic: 53; anoxic: 38) and were significantly slower than the non-flick search patterns (P = 0.016) (95% CI = 2.9, n = flicks: 53; non-flicks: 42) seen at oxic areas of the observation chamber. In anoxic conditions, run and reverse search patterns were 1.6 times more frequent than in oxic conditions (P = 0.040) (95% CI = 2.3, n = oxic: 29; anoxic: 46). In oxic or anoxic conditions there were no differences in swimming search patterns (P = 0.778) (95% CI = 1.4, n = oxic: 13; anoxic: 14).

**Table 1:** Maximum, and mean search pattern speeds (µm s^−1^) and total search pattern numbers (n) identified in oxic and anoxic regions of the observation viewing chamber.

Search pattern	Observation chamber region				Aerobically growncultures	Anaerobicallygrown cultures
	Edge (Oxic)		Center (Anoxic)			
	Chamber	Surface	Chamber	Surface		
Straight swimming	56*, 21 (n = 13)	18*, 14 (n = 7)	46*, 18 (n = 14)	21*, 14 (n = 22)	47*, 32 (n = 9)	65*, 46 (n = 40)
Run-reverse	47*, 21 (n = 21)	35*, 22 (n = 12)	49*, 20 (n = 46)	29*, 15 (n = 9)	76*, 45 (n = 13)	59*, 48 (n = 6)
Combined non-flicks	57*, 25 (n = 25)	35*, 19 (n = 19)	49*, 19 (n = 60)	29*, 14 (n = 31)	76*, 40 (n = 22)	61*, 46 (n = 46)
3-step flicks	37*, 17 (n = 17)	41*, 25 (n = 21)	43*, 20 (n = 38)	25*, 17 (n = 10)	79*, 45 (n = 38)	56*, 46 (n = 5)

* = maximum search pattern speed.

n = number of recordings.

### *Vibrio coralliilyticus* Search Patterns Change from Oxic to Anoxic Environments

**Figure 3:**
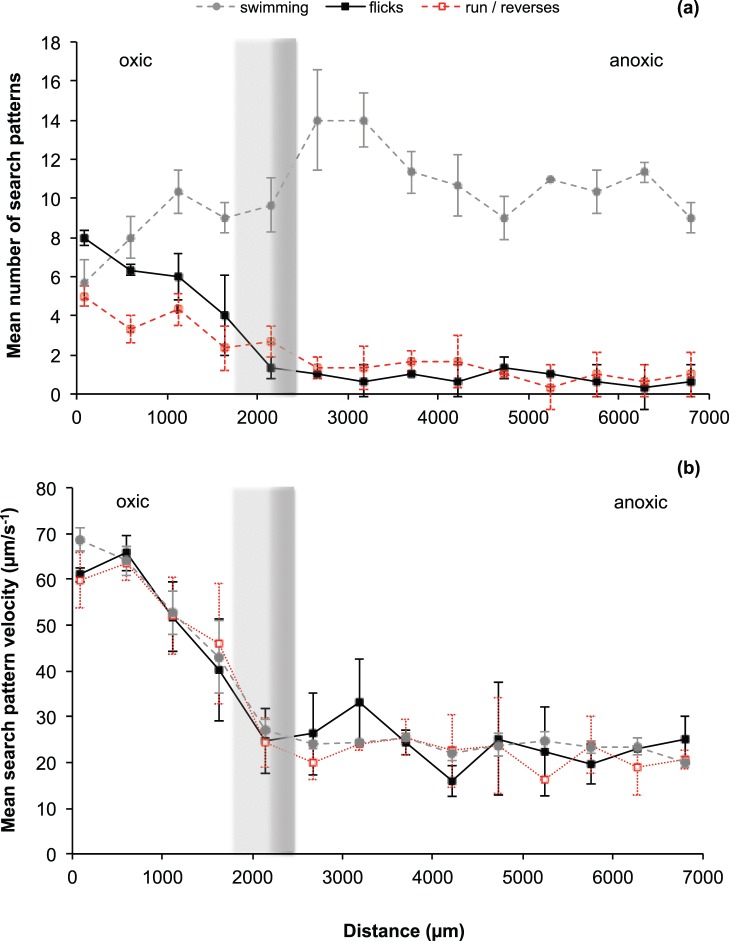
*Vibrio coralliilyticus* search patterns along an oxygen gradient. (a) The mean total number of search patterns seen along the transect line consisting of 40 quadrat boxes (error bars = 95% CI); (b) Mean velocity (µm s^−1^) of search patterns across the transect line (error bars = 95% CI). The location of the grey gradient lines represents the location where the oxygen transition occurs in Fig. 1 (orange and green area).

The chemotactic search pattern behavior of *V. coralliilyticus* changed over the oxic-anoxic interface, identified over a microscope transects line. Three-step flick search pattern numbers decreased 8-fold (P = 0.048), from a mean value of 8 flicks (95% CI = 0.4; n = 24) in oxic environments to a mean value of 0.5 flicks (95% CI = 0.6; n = 3) in anoxic environments. Run and reverse search patterns decreased 5-fold (P = 0.048), from a mean value of 5 run and reverse search patterns (95% CI = 0.51; n = 15) in oxic conditions through to 1 run and reverse (95% CI = 1.13; n = 3) in anoxic conditions. Straight swimming search pattern numbers increased from a mean value of 5.7 straight swimming search patterns (95% CI = 1.2; n = 17) in oxic conditions to a mean value of 9 straight swimming search patterns (95% CI = 0.8; n = 27) in anoxic conditions (Fig. 3a).

Search pattern velocities were shown to decrease 2-fold from oxic to anoxic environments (P = 0.002), from 63.2 µm s^−1^ (95% CI = 2.0, n = 58) to 21.9 µm s^−1^ (95% CI = 3.4, n = 32) respectively, although a large variation in search pattern velocity was identified (Fig. 3). Search pattern velocities remained steady at 23.2 µm s^−1^ from the 2000 µm distance location onwards (P = 0.578, 95% CI = 0.6, n = 400). Velocities did not differ significantly between the three types of search patterns identified, namely the flick, run and reverse and straight swimming (Fig. 3b).

### Aerobic and Anaerobic Controls

Motile cells of *V. coralliilyticus* were observed in cultures grown under both aerobic and anaerobic conditions. Immediate visualization of these cell cultures showed that in aerobically grown cultures, 38 flicks, 13 run and reverses and 9 straight swimming patterns with mean speeds of 45 µm s^−1^ (95% CI = 5.6, n = 38), 45 µm s^−1^ (95% CI  = 8.4, n  = 13) and 32 µm s^−1^ (95% CI  = 0.7, n = 9) respectively, were observed (Table 1). Cultures grown under anaerobic conditions demonstrated 5 flicks, 6 run and reverses and 40 straight swimming search patterns with mean speeds of 46 µm s^−1^ (95% CI = 7.0, n = 5), 48 µm s^−1^ (95% CI = 6.9, n = 6) and 46 µm s^−1^ (95% CI = 3.5, n = 40) respectively (Table 1). In aerobic conditions, flick search patterns were 7.6 times greater than in anaerobic cultures (P = 0.024). In addition, a 2.2 fold increase in run and reverse search patterns (P = 0.010, 95% CI = 8.4, n = 13) and a 4.4 fold decrease (P = 0.003, 95% CI = 6.6, n = 9) in straight swimming search patterns were seen in aerobic conditions compared to anaerobic conditions. Search patterns speeds did not differ across aerobic or anaerobically grown cultures (P = ≥0.05, 95% CI = 2.6, n = 111) (Table 1).

### *Vibrio coralliilyticus* Search Pattern Changes Over Time

**Figure 4:**
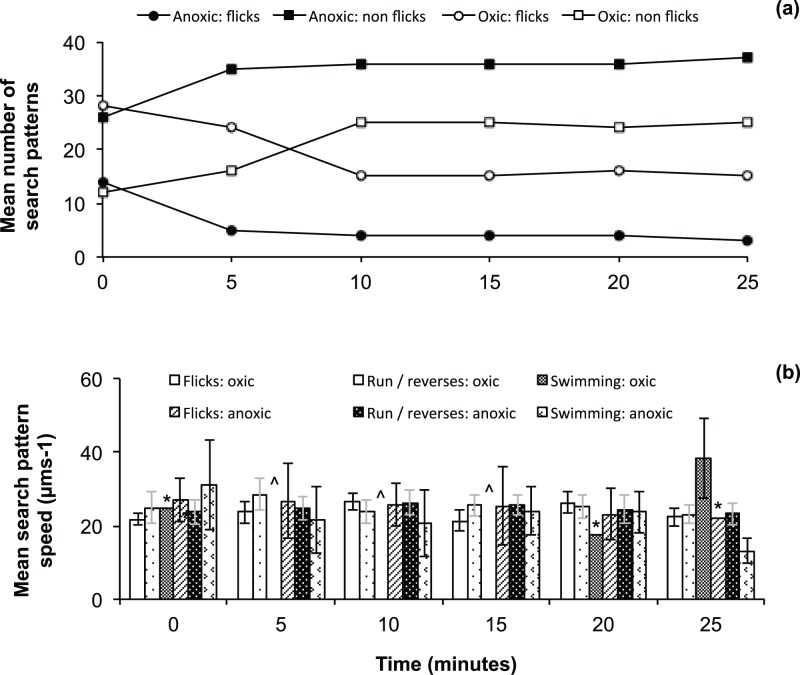
*Vibrio coralliilyticus* search patterns over a 25 minute time series. (a) The mean total number of search patterns seen at the edge of the cover slip and center of the cover slip over a 25 minute time series. The solid squares indicate the number of non-flick search patterns at an anoxic area of the chamber, open squares indicate the number of non-flick search patterns at an oxic area of the chamber, closed circles indicate the number of flicks search patterns at an anoxic area of the chamber, open circles indicate the number of flick search patterns at an oxic area of the chamber; (b) The mean search pattern velocities seen at oxic and anoxic regions of the cover slip chamber over a 25 minute time experiment. * = only 1 search pattern observed so no 95% CI value could be calculated; ? = missing data as no search patterns were identified.

During a 25-minute time series experiment, the search pattern numbers in anoxic conditions leveled off by the 5-minute mark, whereas in oxic conditions, search pattern numbers began to level off from 10 minutes onwards. In anoxic conditions, flick search patterns decreased by 14 times (P = 0.022, 95% CI = 8.0, n = 38), whilst non-flick patterns increased by 8 (95% CI = 3.4, n = 26) over the 25-minute experiment, though this result was not significant (P = 0.230) (Fig. 4a). Straight swimming search patterns remained constant (P = 0.333, 95% CI = 3.1, n = 203). Similarly, in oxic conditions, flick search patterns decreased 1.8 times (P = 0.014, 95% CI = 2.5, n = 120), whilst non-flick search patterns were variable and demonstrated no significant trends (P = ≥0.05, 95% CI = 3.4, n = 122). All search pattern speeds except the straight swimming patterns remained relatively constant at oxic and anoxic conditions over time (Fig. 4b).

## Discussion

Oxygen concentrations can differ between the surrounding water layer and the various microbiomes present in coral organisms, namely the surface mucus layer and coral tissue [17]. During darkness the coral can experience complete hypoxia [26], [27], [28]. As such, coral microorganisms must be able to survive in changing oxygen conditions [14]. We provide evidence that the coral pathogen, *V. coralliilyticus* is capable of utilizing chemotactic search patterns in oxic and anoxic conditions whilst maintaining search pattern velocities across oxic to anoxic conditions. This indicates that regardless of oxygen concentrations *V. coralliilyticus* are capable of remaining motile.

Our results indicate that *V. coralliilyticus* uses the newly discovered cyclic flick search pattern, first found in *V. alginolyticus*, which is morphologically distinct from the run and reverse or run and tumble patterns adopted by *E. coli* [5], [8], [14]. The search pattern behavior identified in oxic and anoxic conditions in *V. coralliilyticus* may be the result of saturated oxic conditions, similar to that seen in *Salmonella typhimurium* and *Escherichia coli* [29], whereby in high concentrations, dissolved oxygen is a repellent and in low conditions (0.25 mM), an attractant [29]. Whilst it is known that the 3-step flick allows for a 90° directional change to relocate to a more suitable nutrient environment [5], [14], it is unknown whether bacteria may use the 90° flick in this search pattern to quickly move away from undesired nutrient patches as often the initial flick is ≥5 µm in length and continues for a distance of ∼ 15 µm [5]. Our work adds to this by showing that the 3-step flick is present in oxygenated environments and not in deoxygenated environments. Determining the critical oxygen concentration for turning off the flick, and why oxygen might be needed provides pathways for further investigating the 3-step flick.

**Figure 5:**
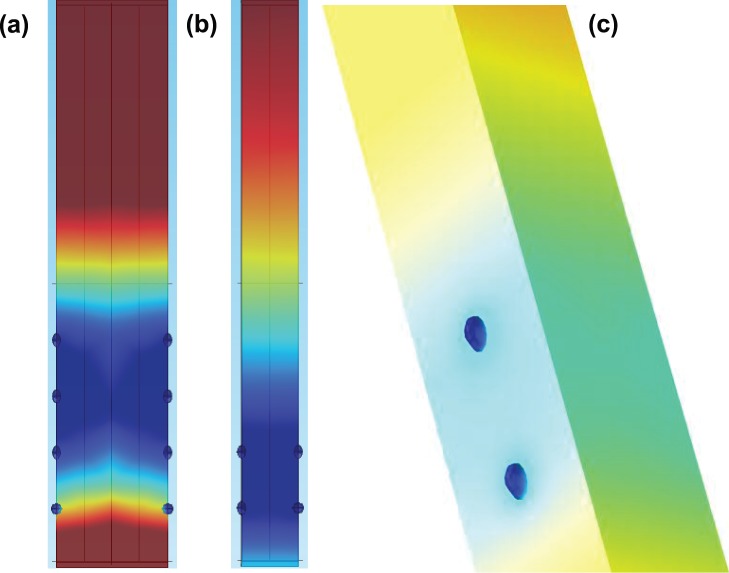
Three-dimensional numerical models of oxygen diffusion microscope slide chamber where oxygen diffuses from the top and the bottom. Blue dots represent bacteria that absorb oxygen as it diffuses past. The columns are 100 µm high (a and b). The relative concentration difference between blue and red is 30 mM. Orthogonalized spacings represent average cell distributions seen in the experiments with (a) for low cell density regions and (b) for cell cluster regions. Real distributions will be heterogenously rather than uniformly distributed. The diagonal in (c) is the rotated volume showing the z axis and the position of the bacteria in that direction.

Changes in search pattern behavior of *V. coralliilyticus* in oxic and anoxic conditions may not be a response to the oxygen concentrations directly, or lack thereof. Instead, as suggested by Armitage [30] bacteria can monitor changes in the electron transfer rate using redox sensors, which are signaled through sensory pathways. This sensory system converts environmental signals into a rotational change of the flagellar motor [31] allowing for cell relocation or revisiting older nutrient patches [32]. All cells possess an ability to sense oxygen and activate these adaptive sensory processes, which are fundamental to survival when oxygen becomes limited [32]. The observation chamber, which is open on two sides, creates an oxygen gradient from the interplay of microbial oxygen consumption and diffusion from the edges (Fig. 5). This simulates the diurnal and nocturnal changes that occur in the surface mucus layer and coral epithelial tissue highlighted in Kühl *et al.* [17]. The behavioral responses arising from this oxygen gradient, illustrates the behavioral complexities of a *V. coralliilyticus* infection, where *V. coralliilyticus* cells remain motile in both oxic and anoxic conditions. With *V. coralliilyticus’s* motility fundamentally linked to infection and establishment in corals [20], knowledge of its behavioral characteristics could provide greater insight into infection rates and mechanisms.

## Supporting Information

Figure S1 A sample video recording of *Vibrio coralliilyticus* cells. The cells tracked can be identified by the open circles, and the specific search pattern trajectories used have been overlaid. The red and yellow lines are flicks, the orange and white lines are straight swimming, and the blue line is a run and reverse. Examples of the 90° flicking events are marked by an X.
(MOV)

Table S1 Experimental number of recordings (n) for *V. coralliilyticus* search pattern preference from oxic to anoxic conditions in Figure 3.
(DOCX)

Table S2 Experimental number of recordings (n) for *V. coralliilyticus* search pattern changes over time in Figure 4.
(DOCX)
