# Supplementary material for: Vibrio coralliilyticus Search Patterns across an Oxygen Gradient
Source: PLoS One. 2013 Jul 10;8(7):e67975. doi: 10.1371/journal.pone.0067975 (PMC3707849; doi:10.1371/journal.pone.0067975)
Supplement: Table S1 — Experimental number of recordings (n) for V. coralliilyticus search pattern preference from oxic to anoxic conditions in Figure 3. (DOCX) [file pone.0067975.s002.docx]

Supplementary Table 1.

| **Search pattern type** | **Number of recordings** | |
| --- | --- | --- |
|  | **Oxic** | **Anoxic** |
| 3-step flick | 24 | 3 |
| Run/ reverse | 15 | 3 |
| Straight swimming | 17 | 27 |
