# Supplementary material for: Vibrio coralliilyticus Search Patterns across an Oxygen Gradient
Source: PLoS One. 2013 Jul 10;8(7):e67975. doi: 10.1371/journal.pone.0067975 (PMC3707849; doi:10.1371/journal.pone.0067975)
Supplement: Table S2 — Experimental number of recordings (n) for V. coralliilyticus search pattern changes over time in Figure 4. (DOCX) [file pone.0067975.s003.docx]

Supplementary Table 2.

| **Time (mins)** | **Number of recordings** | | | | | |
| --- | --- | --- | --- | --- | --- | --- |
|  | **Oxic** | | | **Anoxic** | | |
|  | 3-step flick | Run/ reverse | Straight swimming | 3-step flick | Run/ reverse | Straight swimming |
| 0 | 28 | 11 | 1 | 14 | 22 | 4 |
| 5 | 25 | 16 | 0 | 9 | 25 | 7 |
| 10 | 18 | 23 | 0 | 6 | 29 | 5 |
| 15 | 15 | 25 | 0 | 4 | 25 | 11 |
| 20 | 18 | 21 | 1 | 4 | 17 | 19 |
| 25 | 16 | 21 | 3 | 1 | 30 | 9 |
